# Urchin-Like Ni_2/3_Co_1/3_(CO_3_)_1/2_(OH)·0.11H_2_O for High-Performance Supercapacitors

**DOI:** 10.3389/fchem.2018.00431

**Published:** 2018-09-28

**Authors:** Zi-Min Jiang, Ting-Ting Xu, Cong-Cong Yan, Cai-Yun Ma, Shu-Ge Dai

**Affiliations:** Key Laboratory of Material Physics of Ministry of Education, Zhengzhou University, Zhengzhou, China

**Keywords:** porous, NiCo hydroxides, graphene, composites, supercapacitors

## Abstract

Here, we report our finding in the fabrication of novel porous urchin-like Ni_2/3_Co_1/3_(CO_3_)_1/2_(OH)·0. 11H_2_O (denoted as NC) nanomaterial composed of numerous nanoneedles through an one-step hydrothermal method, which deliveres a high specific capacity of 318 C g^−1^ at a current density of 1 A g^−1^. Moreover, an architectural composite electrode consisting of the porous NC nanoneedles wrapped by reduced graphene oxide (rGO) nanosheets exhibits large specific capacity (431 C g^−1^ at 1 A g^−1^), high rate capability and long cycling life (94% capacity retention after 5,000 cycles at 20 A g^−1^). The presence of rGO in the composite electrode greatly improves the electronic conductivity, providing efficient current collection for fast energy storage.

## Introduction

Supercapacitors (SCs), as an electrochemical energy storage device with high power density, long cycle life and fast charging capability, are considered as one of the most promising energy storage devices (Liang et al., 2017). The most attractive feature of SCs is its fast charging capability with large current density than other energy storage devices (Aricò et al., 2005). The structures and properties of the electrode materials are critical to the performance of the energy storage devices (Shi et al., 2017). Therefore, it has greatly attracted the interest of the researches to develop new electrode materials with excellent specific capacitance and good cycle life (Zhang et al., 2018a; Zhao et al., 2018a).

The rate performance and cycle life of the electrode materials are playing a significant role in the practicality for supercapacitors (Dai et al., 2017). Transition metal compounds such as cobalt hydroxides (Jiang et al., 2018; Long et al., 2018; Yang et al., 2018a), nickel hydroxides (Zhang et al., 2010; Ji et al., 2013; Jiang et al., 2017) with high theoretical capacity have been considered as the most promising positive electrodes for high-performance SCs. Unfortunately, the drawbacks of transition metal materials, such as limited useful cycle life and the poor rate performance, still need further improvement. One of the solutions is to design a nanostructure with large specific surface area, which can enlarge the electrode-electrolyte contact area and improve the effective utilization of the electrode materials, thereby expanding the specific capacitance of the SCs (Liu et al., 2017). Although some novel nanostructures of transition metal compounds have been reported for SCs, such as nanoparticles (Wei et al., 2014), nanowires (Zhao et al., 2018b), nanoflakes (Cao et al., 2007; Pan et al., 2018). However, the rate performance and cycle life of those electrode materials are not satisfactory, the depth of the electrode reaction decreases with the increasing current. In particular, urchin-like nanostructure composed of numerous nanounits have attracted great interest as an unusual group with well-defined nanoneedles structure and shell structure in functional materials (Jin et al., 2018). Their unique structure possess larger surface area and higher pore volume, which are favorable for bringing about more active reaction sites in the electrolyte, the ultra-fine nano-needle structure can also effectively reduce the structural force during charging and discharging, and the electrode material has higher stability and safety.

Recently, some research results show that the composites of nickel and cobalt bimetallic electrode materials show a better cycle stability than the corresponding nickel or cobalt electrode materials because of their complementary advantages and synergistic effects (Chen et al., 2018a; Liu et al., 2018). Nonetheless, how to synthesize such electrode materials with urchin-like nanostructure in a simple and efficient way is still a challenge. In this work, we report our findings in the synthesis and characterization of the unique urchin-like nanostructure with large surface-to-volume ratio. In particular, the NC Co-doping crystal with Co(CO_3_)_x_(OH)_y_ anions possessed one-dimensional chain-like crystal structure was successfully prepared, which delivered a specific capacity of 318 C g^−1^ at a current density of 1 A g^−1^. Moreover, we successfully prepared the NC/rGO nanocomposite by introducing rGO, which exhibited enhanced electrochemical performance compared to that of the NC nanomaterial.

## Experimental section

### Preparation of NC nanomaterial

All chemical reagents in this work were used as received without further purification. The urchin-like NC nanomaterial was synthesized by one-step hydrothermal method. First, 1 mmol of NiCl_2_·6H_2_O (aladdin, 99%), 3 mmol of CH_4_N_2_O (aladdin, 99%) and 0.5 mol of CoCl_2_·6H_2_O were first dissolved in 10 mL of deionized water in the Teflon containers by stirring for 30 min to form homogeneous solution. Then the autoclave was sealed and maintained at a temperature of 100°C for 12 h. After natural cooling to room temperature, the samples were taken out, rinsed with deionized water until pH is close to 7, and dried under vacuum at 70°C overnight.

### Preparation of NC/rGO nanomaterials

The NC/rGO composite was synthesized using a hydrothermal method similar to the synthesis of NC. 1 mmol of NiCl_2_·6H_2_O (aladdin, 99%), 3 mmol of CH_4_N_2_O (aladdin, 99%) and 0.5 mmol of CoCl_2_·6H_2_O were first dissolved in 10 mL of GO solution (2 mg mL^−1^) in the Teflon container by stirring 30 min to form homogeneous solution. Then the autoclave was sealed and maintained at a temperature of 100°C for 12 h. After natural cooling to room temperature, the samples were taken out, rinsed with deionized water until pH is close to 7, and dried under vacuum at 70°C overnight.

### Material characterization

The phase structures of the samples were characterized by X-ray diffraction (XRD) analysis (XRD, PA National X′ Pert Pro with Cu Kα radiation), The microstructure and morphology of NC nanomaterials were characterized using field emission scanning electron microscopy (Zeiss, sigma300) and high-resolution transmission electron microscopy (HRTEM, JEOL, JEM-2100) with energy dispersive X-ray spectrometry (EDS). The nitrogen adsorption-desorption isotherm measurement of the sample was performed using a ASAP2420-4MP. The specific surface area was obtained by the Brunauer-Emmett-Teller (BET) method. The element atomic ratio analysis were characterized by inductively coupled plasma mass spectrometry (ICP MS) using a Agilent 7700.

### Electrochemical measurement

The as-prepared NC and NC/rGO electrodes were used as the working electrodes with a platinum sheet electrode and a Ag/AgCl (prefilled with 4 M KCl aqueous solution saturated with AgCl) reference electrodes (Gogotsi, 2014), respectively. The electrochemical tests were performed using a CHI760 electrochemical workstation. Cyclic voltammetry (CV), galvanostatic charge-discharge (GCD) and electrochemical impedance spectroscopy (EIS) tests were carried out using a three-electrode configuration in 2 M KOH aqueous solution at room temperature. For the working electrode, a mixture containing of 80 wt% electroactive material, 10 wt% Super P Li (TIMCAL Graphite and Carbon) powders, and 10 wt% polytetrafluoroethylene (PTFE) binder was well mixed to prepare working electrodes, and then rolled with the assistance of ethanol to form a uniform film with a typical area mass loading of approximately 1.5 mg cm^−2^, and dried under vacuum at 80°C for 12 h, then pressed it between two nickel foams. The cyclic voltammograms were acquired in a potential range of 0–0.5 V at different scan rates, the charge-discharge processes were performed by cycling the potential from 0 to 0.45 V at different current densities in a 2 M KOH aqueous electrolyte, and the EIS was performed in the frequency range from 0.01 Hz to 100 k Hz under open-circuit condition. The specfic capacity Q (C g^−1^) of the battery-type electrodes was calculated as follows (Mai et al., 2013):

(1) $$\begin{array}{r} Q=i_{m}\Delta t \end{array}$$

where *i*_*m*_ = *I/m* (A g^−1^) is the current density, *I* is the current, *m* is the mass of the active electrode material and Δt is the discharge time.

## Results and discussion

**Figure 1:**
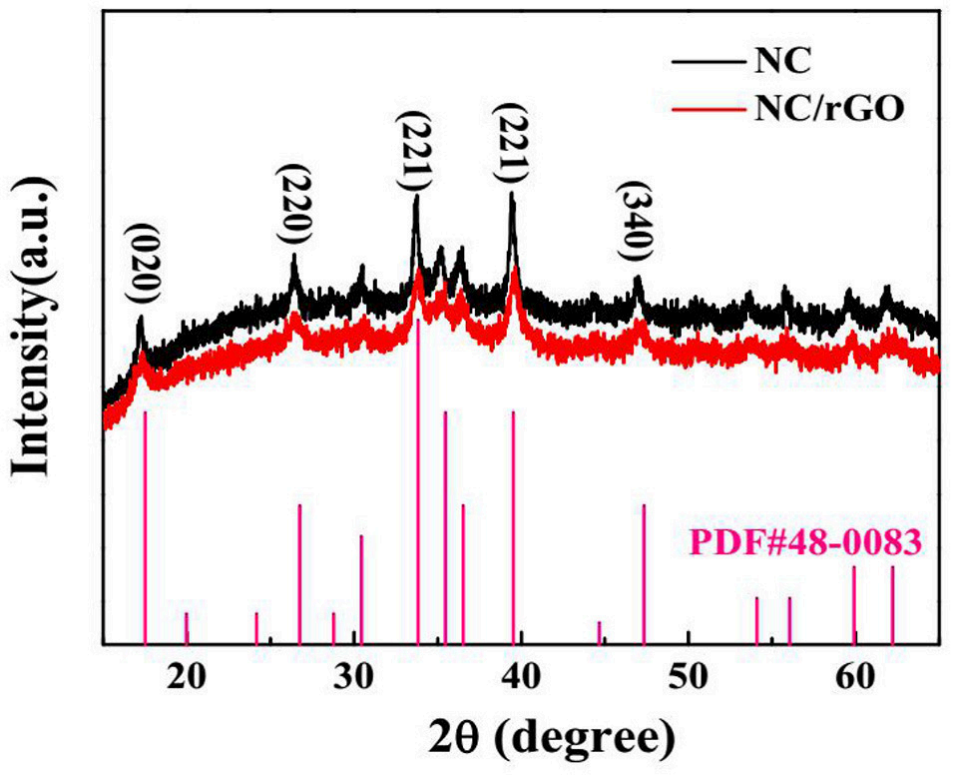
XRD patterns of NC and NC/rGO composite.

It is well known that the polymorphs of nickel hydroxides depend on the hydrothermal reaction conditions such as the ratio of precursors, reaction temperature and time (Li et al., 2016). By tuning the initial mole ratio of reactants, we synthesized the urchin-like NC nanomaterials by a simple one-step solvent-thermal method. The XRD pattern of the NC (black curve) sample is illustrated in Figure 1. All diffraction peaks of the XRD pattern can be indexed to orthorhombic Co(CO_3_)_1/2_(OH)·0.11H_2_O (JCPDS NO. 48-0083). The strong diffraction peaks centered at 2θ = 17.2°, 26.4°, 33.8°, 39.4°, and 46.9°, attributed to (0 2 0), (2 2 0), (2 2 1), (2 3 1), and (3 4 0), respectively. And the XRD pattern of NC/rGO (red curve) sample is extremely similar to the NC pattern, and no other obvious peaks are detected. The reaction mechanism may be as follows (Wei et al., 2017):

(1) $$CO{\left(NH_{2}\right)}_{2}+H_{2}O=CO_{2}+2NH_{3}$$

(2) $$\begin{array}{r} CO_{2}+H_{2}O=2H^{+}+C{O_{3}}^{2-} \end{array}$$

(3) $$\begin{array}{r} NH_{3}+H_{2}O=N{H_{4}}^{+}+OH^{-} \end{array}$$

(4) $$\begin{array}{l} {CO_{3}}^{2-}+2A^{2+}+0.22H_{2}O+OH^{-}=2A{\left(CO_{3}\right)}_{0.5}(OH)\\ \;\;\;\;\;\;\;\;\;\;\;\;\;•0.11H_{2}O \end{array}$$

In the chemical reaction system, firstly, CO(NH_2_)_2_ combined with H_2_O to produce CO_2_ and NH_3_ (equation 1). Then, CO_2_ and NH_3_ combined with H_2_O immediately to produce ${\text{CO}}_{3}^{2-}$, OH^−^ and NH^4+^ (equations 2 and 3), respectively. Finally, ${\text{CO}}_{3}^{2-}$ and OH^−^ combined with A^2+^ (Ni^2+^ or Co^2+^) to form the sample (equation 4).

**Figure 2:**
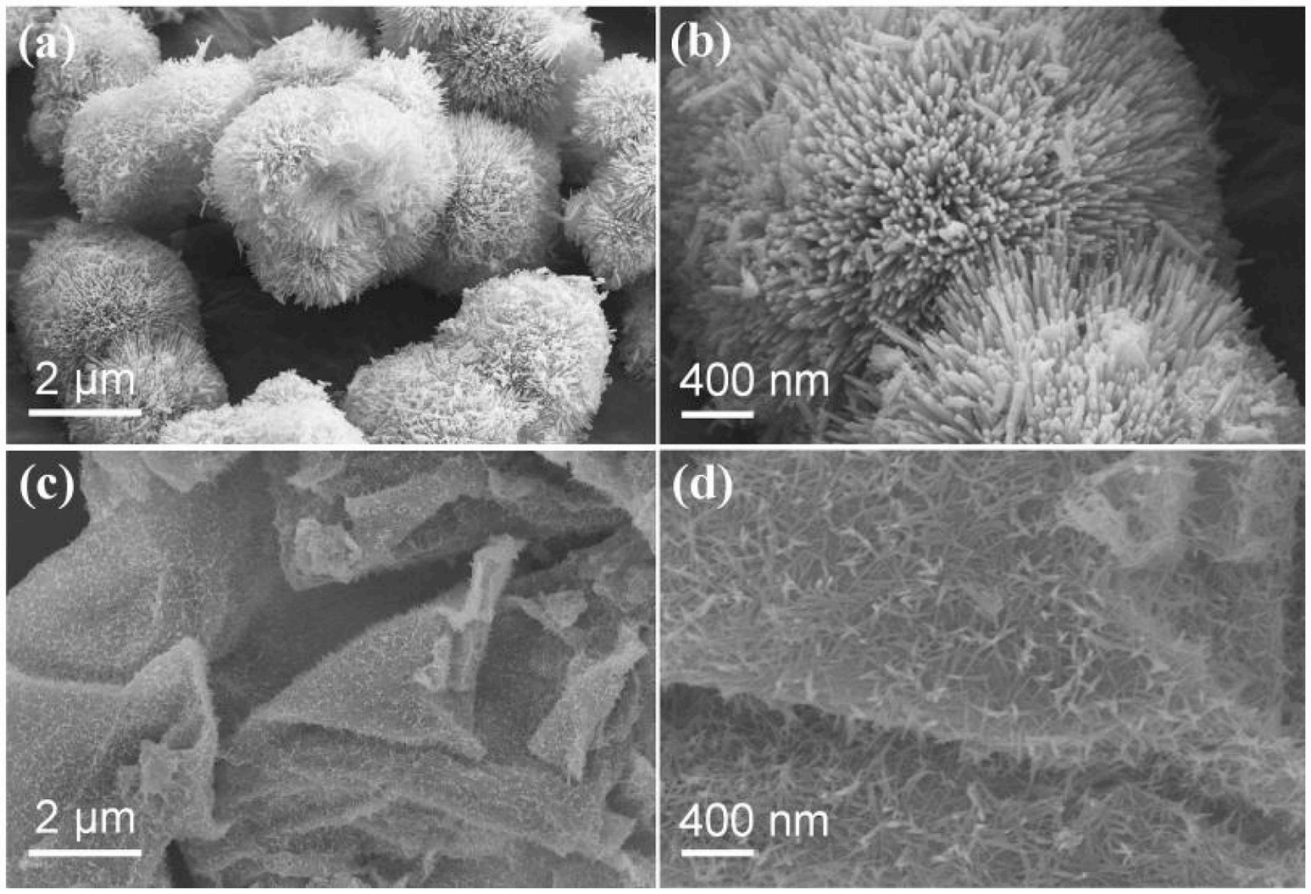
Scanning electron microscopy (SEM) images of as-synthesized NC and NC/rGO nanomaterials. **(a,b)** urchin-like NC nanoball, **(c,d)** as-synthesized NC/rGO composite nanosheets.

**Figure 3:**
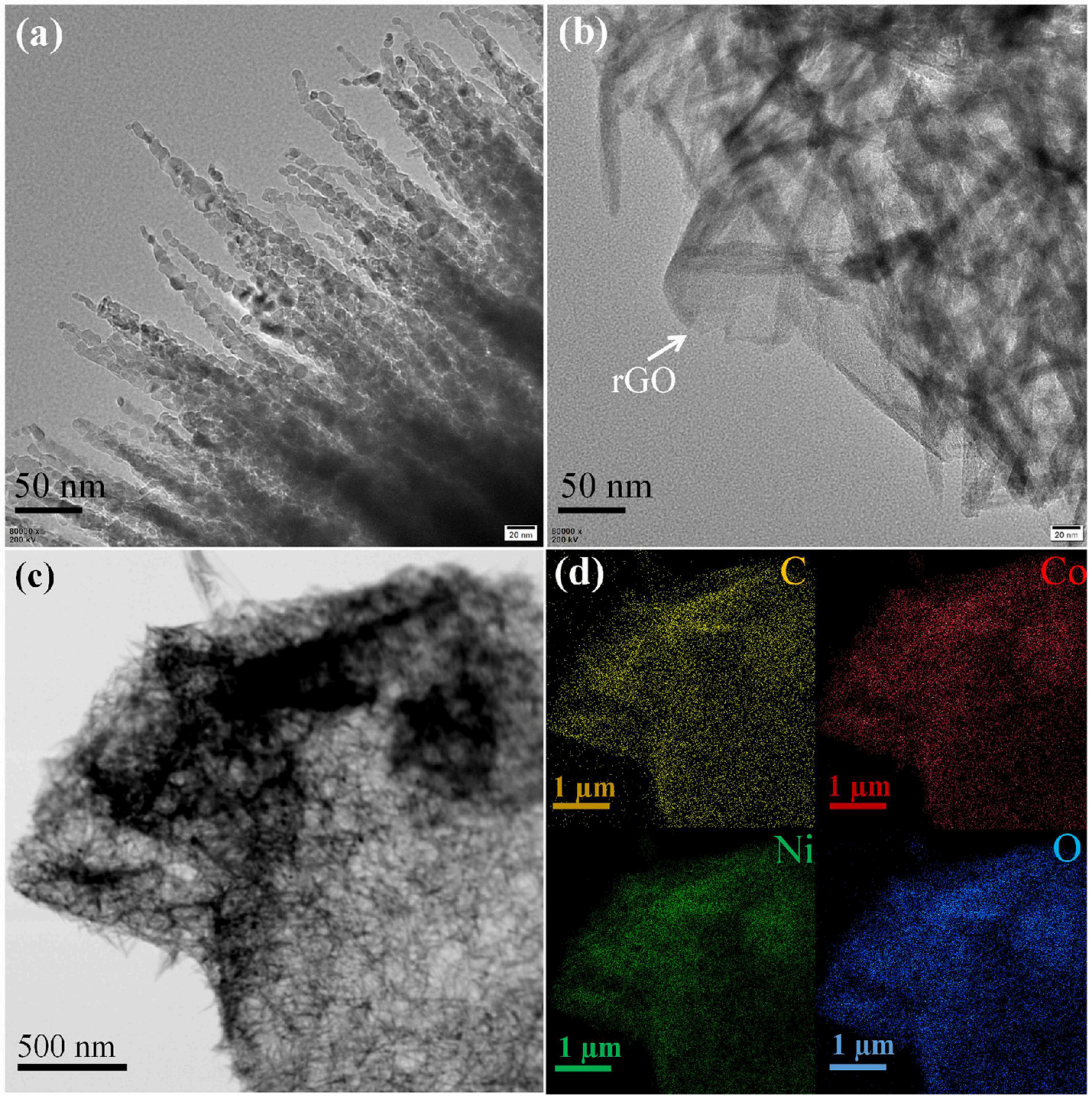
TEM image of NC and NC/rGO composite. **(a)** high magnification of NC urchin-like nananeedles, **(b)** high magnification of NC/rGO composite nanosheets, **(c)** the bright field TEM image of NC/rGO composite nanosheets. **(d)** TEM-EDX mapping images NC/rGO composite nanosheets.

**Figure 4:**
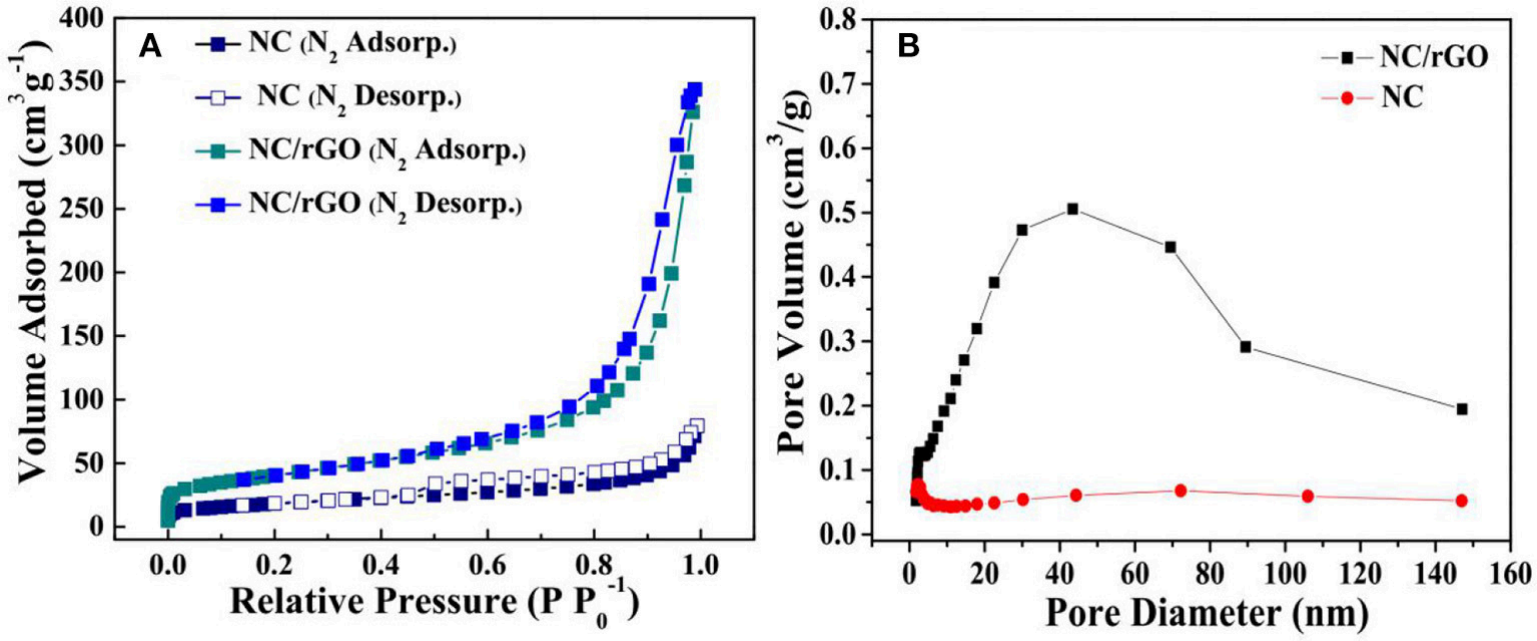
**(A)** N_2_ adsorption-desorption isotherms and **(B)** corresponding pore size distribution plot of urchin-like NC nanoball and NC/rGO composite nanosheets.

SEM was performed to examine the morphology and structure of the as-synthesized samples. Figure 2a shows the low-magnification SEM image of as-synthesized NC nanomaterial, which appears to be thorn balls constructed of numerous nanoneedles, and similar to the urchin-like microspheres. In Figure 2b, the high magnification SEM image reveals that the diameters of those nanoneedles are around 10–20 nm. The morphology evolution mechanism of urchin-like NC may be as follows: firstly, the ultrashort nanoneedles *in situ* grow on the surface of the solid core as the self-generated templet without crack and finish solid core growth. Owing to the increasing of the amount of NH_3_ and ${\text{CO}}_{3}^{2-}$, NH_3_ holds the metal ions moving to the top of the ultrashort nanoneedles with high surface energy and generates precipitate, which leads to the preferred growth of product at the top of short nanoneedles and makes the short nanoneedles slowly into the long nanoneedles. The SEM images of the NC/rGO samples are shown in Figures 2c,d, and the morphology of the composite is nanosheets. These NC nanoneedles are distributed uniformly in the rGO nanosheets, and numerous nanosheets over-lap together to form a irregular three-dimensional architecture. Notably, this unique nanostructure with large surface is favorable for efficient and fast transport of the electrolyte to the surface of the active materials, thus improving the effective utilization of the active material. The high magnification TEM images (Figures 3a,b) show the clear morphology of the nanoneedles in the NC and NC/rGO materials. These nanoneedles with a diameter of 10~20 nm (Figure 3a) build an unique urchin-like nanostructure, which greatly shorts the electron transport channel, and improves the conductivity of the electrodes. In Figure 3b, the NC nanoneedles are attached to the rGO nanosheets, forming a composite nanostructure. However, the urchin-like suffered from the deficiency of those shells, the internal active material could not make full contact with the electrolyte and then affect the performance of the whole electrode. Fortunately, the addition of rGO nanosheet overcomes this shortcoming, these nanoneedles distributed uniformly on the graphene nanosheets, which provides an effective pathway for charge transpor, weakens the polarization phenomenon at high current, and further improves the effective utilization of the active material and the rate performance of the electrodes. Figures 3c,d shows the bright field TEM image and mapping images of the NC/rGO composite, which clearly reveals that the NC nanoneedles are distributed homogeneously in the rGO nanosheets. The molar ratio of Ni:Co is determined to be 2:1 based on the result analysis, which is similar to the results of ICP-MS (~70.03:36.89, Table 1), and close to the mole ratio of adding nickel and cobalt sources, proving that urchin-like NC nanomaterial possesses a molecular formula of NC. To investigate the porosity and surface area of as-prepared samples, N_2_ adsorption-desorption isotherms of NC and NC/rGO conducted at 77.350 K were investigated and are displayed in Figure 4A. Through Brunauer-Emmett-Teller (BET) analysis, the surface areas of NC and NC/rGO composite were identified as 62.55 m^2^g^−1^ and 138.27 m^2^g^−1^, respectively. In addition, the pore size distribution curves were also investigated via using Barrett-Joyner-Halenda (BJH) method (Figure 4B). For NC, no obvious peaks arise in the entire range of pore size distribution curve. For NC/rGO, an obvious peak at around 40~50 nm in the pore size distribution curve was observed, which was attributed to the rGO sheets. These nanosheets provide a lot of attachment points for the growth of nanoneedles through the hydrothermal treatment. Higher surface area offers abundant reaction sites and enhances the specific capacity of the NC/rGO composite electrodes.

**Table 1:** Results of ICP-MS for NC-1 and NC-2 and NC-3.

**SOLUTION CONCENTRATION (MG L**^**−1**^**)**			
**Sample name**	**Ni**	**Co**	**Atomic ratio of Ni:Co**
NC-1	8.3046	4.3271	69.56:36.71
NC-2	8.4231	4.3657	70.55:37.04
NC-3	8.3546	4.3520	69.98:36.92

**Figure 5:**
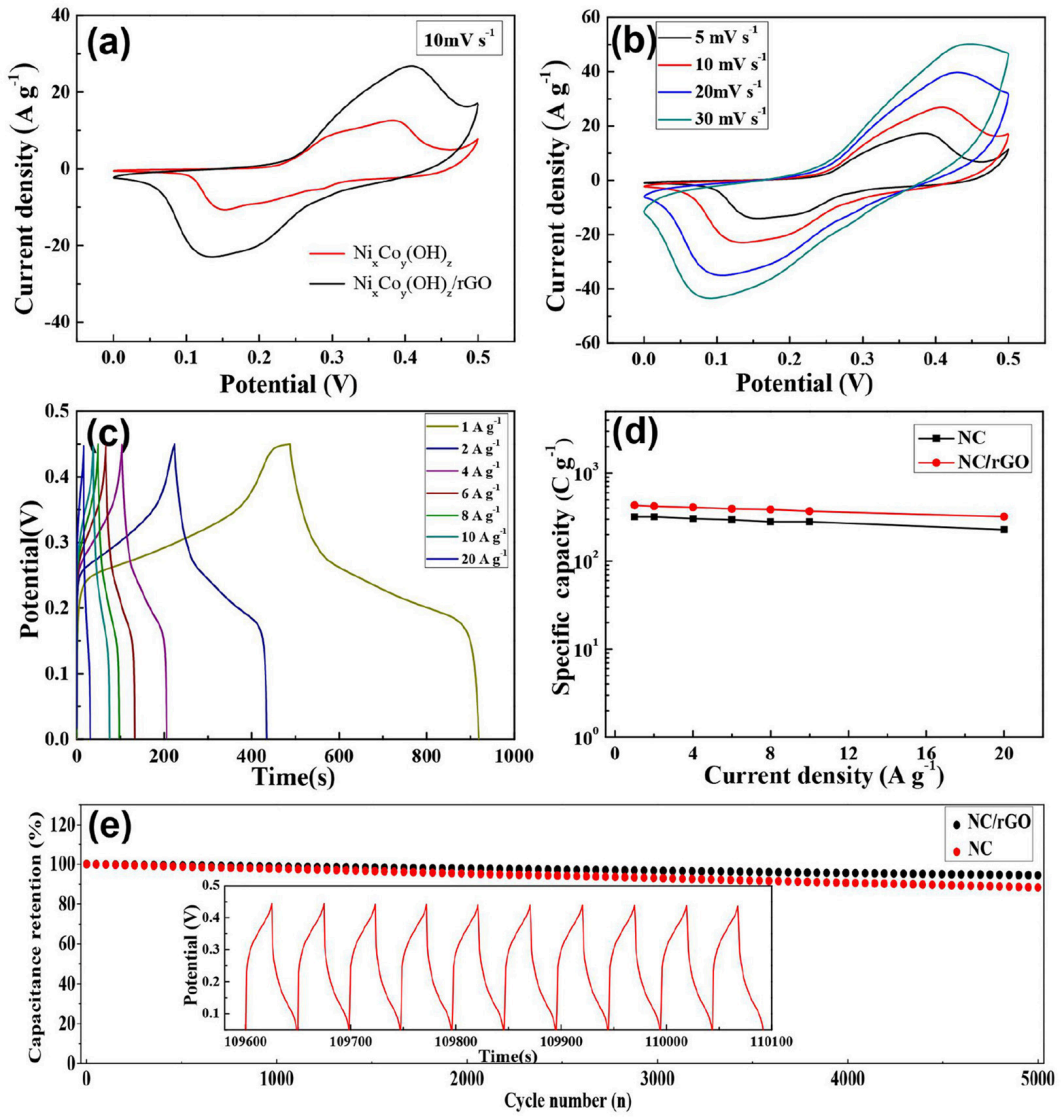
**(a)** CV curves of NC nanoball and NC/rGO composite nanosheets at a scan rate of 10 mV s−1. **(b)** CV curves of NC/rGO composite nanosheets at at various scan rates. **(c)** GCD curves of NC/rGO composite nanosheets at different current densities. **(d)** The specific capacity of NC nanoball and NC/rGO composite nanosheets at different current densities. **(e)** Long-term cycle performance of NC/rGO composite electrode and NC electrode at a current density of 20 A g^−1^ (corresponding charge/discharge curves of the last 10 cycles for the 5000 cycles is shown in inset).

The electrochemical performance of the NC microspheres and NC/rGO nanocomposite were first evaluated with a three-electrode configuration in 2 M KOH aqueous electrolyte, the results are shown in Figures 5a–d and Figure S1. For comparison, the electrochemical performance of NC and NC/rGO electrodes in the potential range between 0 and 0.5 V (versusAg/AgCl) at a scan rate of 10 m V s^−1^ are demonstrated in Figure 5a. Evidently, the NC/rGO electrode exhibits a higher current density and larger increment in the CV integrated area than NC electrode, which indicates an obvious enhanced of electrochemical reaction activity and charge storage, suggesting the improvement of conductivity and electrochemical performance. Figure 5b displays the CV curves of the NC/rGO composite electrode at different scan rates; all the CV curves exhibit a pair of redox peak, which indicates that the energy storage in our electrodes appears to be battery-type materials. The corresponding CV curves of NC microspheres was also collected and shown in Figure S2. It is clear that all of the plateaus at around 0.05–0.15 V, which correspond to the redox processes. Compared with the CV curves of NC, all of the CV curves of the NC/rGO composite electrode turn into smooth, indicating that the rGO greatly improved the conductivity of the composite electrodes.

The specific capacities of the NC and NC/rGO electrodes were calculated based on the GCD curves, and the corresponding GCD curves of NC and NC/rGO electrodes are shown in Figure S2 and Figure 5c, respectively. As shown in Figure 5d, the NC electrode delivered a specific capacity of 318 C g^−1^ at a current density of 1 A g^−1^, and retained ~71% (228 C g^−1^) of the capacity when the current density was increased from 1 to 20 A g^−1^. In comparison, the NC/rGO electrode exhibted a specific capacity of 431 C g^−1^ at a current density of 1 A g^−1^, and retained ~74% (320 C g^−1^) of capacity, which is higher than that of the NC electrode. The obvious discharge platform further proves the Faraday energy storage mode, demonstrating that the composite electrodes possess battery-like effect in the charge and discharge process (Yang et al., 2018b). Besides, the specific capacity of the NC/rGO were higher than other transition metal compounds reported in literature, such as Co_0.45_Ni_0.55_O/RGO nanocomposites (411 C g^−1^ at a current density of 1 A g^−1^)(Xiao and Yang, 2012), NiCo_2_-RGO nanocomposites (417 C g^−1^ at a current density of 1 A g^−1^)(Wang et al., 2011), NiCo_2_O_4_ nanocomposites (296 F g^−1^ at a current density of 1 A g^−1^)(Xiao and Yang, 2011). The good electrochemical performance of NC/rGO composite nanomaterial may be attributed to its unique morphology and crystal structure. First, the self-contained OH^−^ and the one-dimensional chain-like crystal structure unit increase the electrode material ionic conductivity. Second, the porous composite nanostructure has large specific surface area of 138.27 m^2^g^−1^, which greatly increases the effective area between active material and electrolyte, and facilitates more effective exposure of surface active sites for fast Faradic redox reaction. Third, the rGO nanosheets provides a great specific area for the NC nanoneedles growth, constructing a three-dimensional conducting network, which ensures the excellent rate performance. Cycling life is an important property for the SCs. The NC/rGO composite electrode could still retain ~94% of the initial capacity over 5,000 cycles at a high current density of 20 A g^−1^, which is higher than the NC electrode (~88% of the initial capacity), revealing its good cycling stability (Figure 5e). Furthermore, the cycling stability of the NC/rGO in 2 M aqueous KOH aqueous solution using a typical three-electrode cell were superior to many transition metal compounds electrode materials reported in literature, such as Co(OH)_2_ nanosheets (89.1% capacitance retention after 5,000 cycles at 20 A g^−1^) (Chen et al., 2018b), Co-doped α-Ni(OH)_2_/RGO nanosheet (87.9% capacitance retention after 1,000 cycles at 10 A g^−1^) (Zhang et al., 2018b), Ni(OAc)_2_/Co(NO_3_)_2_ (91.5% capacitance retention after 1,000 cycles at 5 A g^−1^) (Wei et al., 2018), illustrating the excellent capacity and long-term electrochemical stability of the NC/rGO composite electrode.

## Conclusion

In summary, a novel porous NC nanomaterial with urchin-like architecture has been successfully prepared by one-step hydrothermal process. When tested in a three-electrode configuration in 2 M KOH aqueous electrolyte, it is found that the NC nanomaterial exhibits a high specific capacity of 318 C g^−1^ at 1 A g^−1^. Moreover, a porous NC/rGO composite electrode was also successfully prepared, which exhibited good specific capacity (431 C g^−1^ at 1 A g^−1^), great rate capability (~74% capacity retention) and long cycling life (94% capacity retention over 5,000 cycles), demonstrating its promising potential for the high-performance supercapacitors.

## Author contributions

Z-MJ carried out the material preparation and electrochemical test; T-TX and S-GD carried out and analyzed the XRD, SEM, and TEM analysis; Z-MJ wrote the paper and all authors discussed the results; Z-MJ, T-TX, C-CY, C-YM and S-GD revised the manuscript; T-TX attained the main financial support for the research and supervised all the experiments.

### Conflict of interest statement

The authors declare that the research was conducted in the absence of any commercial or financial relationships that could be construed as a potential conflict of interest.
