# Supplementary material for: Urchin-Like Ni2/3Co1/3(CO3)1/2(OH)·0.11H2O for High-Performance Supercapacitors
Source: Front Chem. 2018 Sep 28;6:431. doi: 10.3389/fchem.2018.00431 (PMC6172363; doi:10.3389/fchem.2018.00431)
Supplement: Supplementary file 1 [file Table_1.DOC]

**Urchin-like Ni_2/3_Co_1/3_(CO_3_)_1/2_(OH)·0.11H_2_O for high-performance supercapacitors**

Zi-Min Jiang, Ting-Ting Xu*, Cong-Cong Yan, Cai-Yun Ma, Shu-Ge Dai*

Key Laboratory of Material Physics of Ministry of Education, Zhengzhou University, Zhengzhou 450052, China;

*Corresponding author

School of Physics and Engineering, and Key Laboratory of Material Physics of Ministry of Education, Zhengzhou University, Zhengzhou 450052, PR China

E-mail addresses: [xutt@zzu.edu.cn (T. Xu);](mailto:xutt@zzu.edu.cn;) [shugedai@zzu.edu.cn](mailto:shugedai@zzu.edu.cn) (S. Dai)


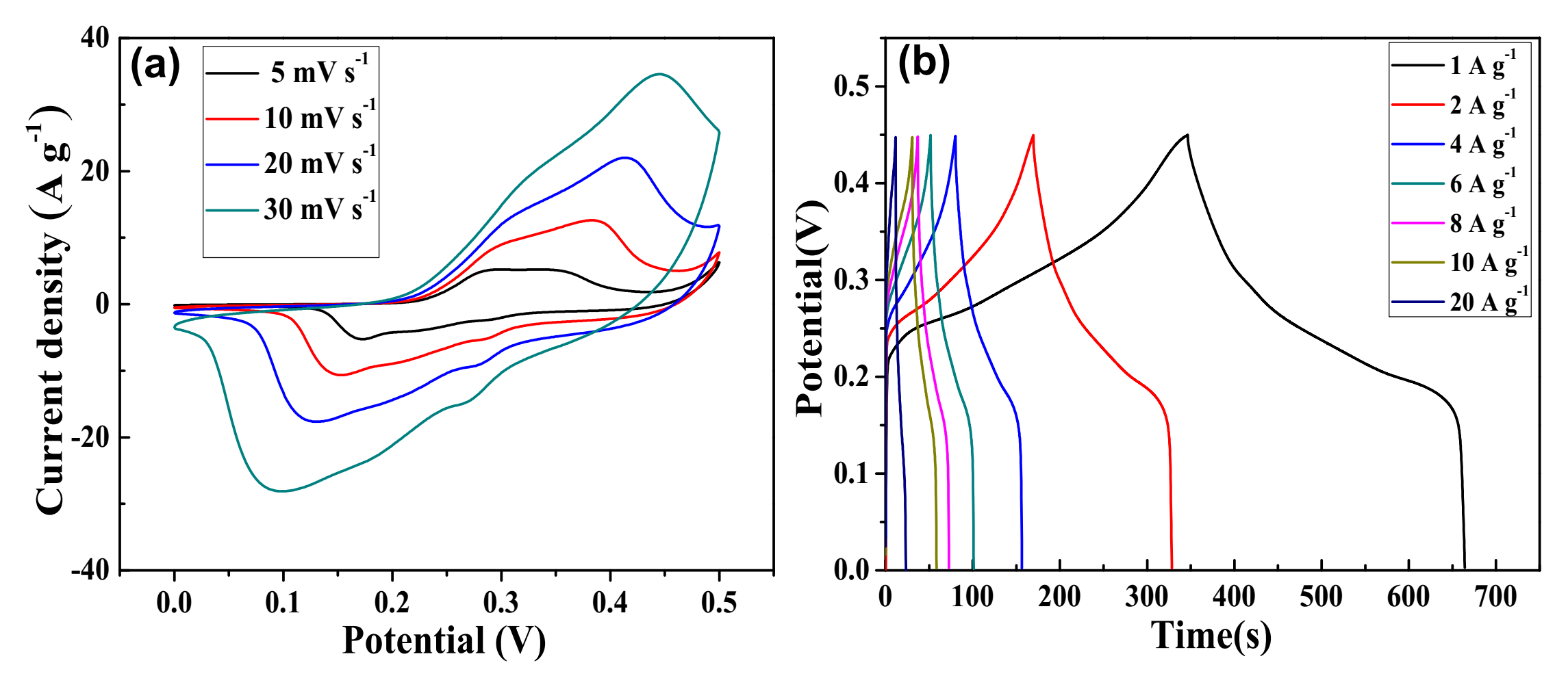


**FIGURE S1.** (a) CV curves of as-synthesized NC nanoball at different scan rates. (b) GCD curves of as-synthesized NC nanoball.


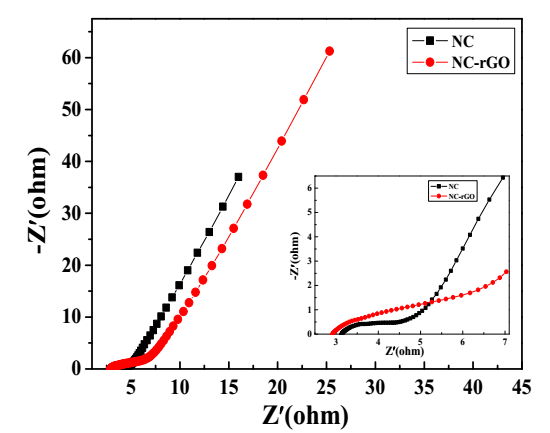


**FIGURE S2.** Nyquist plots of the NC nanoball and NC/rGO composite nanosheets. (the inset is an enlarged view of the Nyquist curves).
